# Effects of cholesterol content on activity of P-glycoproteins and membrane physical state, and consequences for anthelmintic resistance in the nematode *Haemonchus contortus*

**DOI:** 10.1051/parasite/2019079

**Published:** 2020-01-14

**Authors:** Mickaël Riou, Fabrice Guégnard, Yves Le Vern, Isabelle Grasseau, Christine Koch, Elisabeth Blesbois, Dominique Kerboeuf

**Affiliations:** 1 INRAE, UE-1277 Plateforme d’infectiologie expérimentale (PFIE), Centre de Recherche Val de Loire 37380 Nouzilly France; 2 INRAE, Université de Tours, UMR-1282 Infectiologie et Santé Publique (ISP), Centre de Recherche Val de Loire 37380 Nouzilly France; 3 INRAE, CNRS, HARAS NATIONAUX, IFCE, Université de Tours, UMR-0085 PRC Physiologie de la Reproduction et des Comportements, Centre de Recherche Val de Loire 37380 Nouzilly France

**Keywords:** Parasite, Eggshell, MβCD, Fluidity, Cholesterol, P-glycoproteins, Resistance, Anthelmintics

## Abstract

Eukaryote plasma membranes protect cells from chemical attack. Xenobiotics, taken up through passive diffusion, accumulate in the membranes, where they are captured by transporters, among which P-glycoproteins (Pgps). In nematodes such as *Haemonchus contortus*, eggshells and cuticles provide additional protective barriers against xenobiotics. Little is known about the role of these structures in the transport of chemical molecules. Pgps, members of the ABC transporter family, are present in eggshells and cuticles. Changes in the activity of these proteins have also been correlated with alterations in lipids, such as cholesterol content, in eggshells. However, the cellular mechanisms underlying these effects remain unclear. We show here that an experimental decrease in the cholesterol content of eggshells of *Haemonchus contortus*, with Methyl-beta-CycloDextrin (MβCD), results in an increase in membrane fluidity, favouring Pgp activity and leading to an increase in resistance to anthelmintics. This effect is modulated by the initial degree of anthelminthic resistance of the eggs. These results suggest that eggshell fluidity plays a major role in the modulation of Pgp activity. They confirm that Pgp activity is highly influenced by the local microenvironment, in particular sterols, as observed in some vertebrate models. Thus, eggshell barriers could play an active role in the transport of xenobiotics.

## Abstract (fr)

Les membranes plasmiques des eucaryotes protègent les cellules contre les attaques chimiques. Les xénobiotiques, absorbés par diffusion passive, s’accumulent dans les membranes où ils sont capturés par des transporteurs, parmi lesquels les glycoprotéines P (Pgp). Chez les nématodes, les coques des œufs et les cuticules constituent des barrières de protection supplémentaires contre les xénobiotiques. On en sait peu sur le rôle de ces structures dans le transport des molécules chimiques. Les Pgp, membres de la famille des transporteurs ABC, sont présents dans les coques et les cuticules. Des changements dans l’activité de ces protéines ont également été mis en corrélation avec des altérations des lipides, tels que la teneur en cholestérol, des coques des œufs. Cependant, les mécanismes cellulaires sous-jacents à ces effets restent flous. Nous montrons ici que la diminution expérimentale de la teneur en cholestérol des coques des œufs d’*Haemonchus contortus*, avec la méthyl-beta-cyclodextrine (MβCD), entraîne une augmentation de la fluidité membranaire favorisant l’activité des Pgp et une augmentation de la résistance aux anthelminthiques. Cet effet est modulé par le degré initial de résistance aux anthelminthiques des œufs. Ces résultats suggèrent que la fluidité de la coque joue un rôle majeur dans la modulation de l’activité des Pgp. Ils confirment que l’activité des Pgp est fortement influencée par le microenvironnement local, en particulier les stérols, comme observé dans certains modèles de vertébrés. Ainsi, les barrières de coques des oeufs pourraient jouer un rôle actif dans le transport des xénobiotiques.

**Abbreviations**
ABC: ATP-binding cassette
ATP: Adenosine TriPhosphate
DPH: 1,6-DiPhenyl-1,3,5-Hexatriene
HcR: *Haemonchus contortus* Resistant
HcS: *Haemonchus contortus* Susceptible
*I*//: Parallel intensity
*I*⊥: Perpendicular intensity
MβCD: Methyl-beta-CycloDextrin
MDR: MultiDrug Resistance
Pgp: P-glycoprotein
R123: Rhodamine 123
TBZ: ThiaBendaZole

## Introduction

Gastrointestinal nematodes include *Haemonchus contortus*, a highly pathogenic parasite infecting small domestic ruminants [25, 64, 80]. The prophylactic treatment of parasitic gastroenteritis relies mainly on the use of anthelmintics. However, the efficacy of anthelmintics against nematodes is compromised by the emergence of resistant parasites [40, 42, 44, 60]. Resistance to all groups of anthelmintics (benzimidazoles, imidazothiazoles, tetrahydropyrimidines and avermectins) has been observed in many studies [40, 44, 65]. Anthelmintic resistance involves several cellular mechanisms. Both specific anthelmintic resistance, for example mutation of β-tubulin, the target of thiabendazole [5, 41, 42], and non-specific mechanisms have been described. In eukaryotes, the MDR genes and MDR protein activity are responsible for the development of resistance to drugs in tumour cells [1, 37, 43, 72]. The MDR system includes P-glycoprotein membrane “pumps” (Pgps) and multidrug resistance-associated proteins (MRP). These two transmembrane proteins are members of the ATP-binding cassette (ABC) superfamily of transporters, playing key roles in the transport of xenobiotics [1, 36, 71].

Eukaryote cells are protected against chemical attack by their plasma membranes [73]. Many drugs and other xenobiotic molecules are lipophilic and enter the cell membranes primarily by passive diffusion (“passive influx”), which depends on solubilisation in lipids [50]. Then, xenobiotics that accumulate in the membranes are supported by membrane transporters [1, 83]. The transport of xenobiotics thus depends on both the hydrophobicity of cell membranes and on the activity of membrane pumps [10, 11, 59]. These pumps have been implicated in cellular detoxification processes in various eukaryotic systems [3]. They are modulated by the membrane environment [9, 50, 56, 58]. Among these pumps, the overexpression of Pgp confers resistance to xenobiotics in many biological systems, mainly in tumour cells resistant to chemotherapy but also in nematodes resistant to anthelmintics [2, 18, 28].

Transmembrane transport of drugs is modulated by the biochemical composition of the membrane. Qualitative or quantitative changes in membrane lipids modify the properties of cell membranes [58]. Lipids, including cholesterol and phospholipids, play an important role in the passive diffusion of xenobiotics and Pgp activity [17, 68]. Changes in membrane properties directly affect the accessibility of xenobiotic molecules to Pgp. Moreover, cholesterol interacts with phospholipids and proteins, stabilising their movement in the membrane [73] and affecting the activity of many membrane proteins, including receptors, channels, and Pgp [6, 38, 76]. Membrane properties are altered by movements of molecules that determine fluidity, and this depends largely on cholesterol concentration in vertebrate cells [39, 74]. Riou et al. and Rothnie et al. reported significant modulations of Pgp activity, respectively, in tumour cells and nematode isolates after an experimental decrease in cholesterol content [66, 70]. Riou et al. showed that the increase in resistance to anthelmintics observed during egg embryonation resulted from changes in Pgp activity in response to alterations in the membrane environment [67]. However, the biochemical/biophysical mechanisms underlying these effects remain unclear [66, 67]. Hypotheses for a role of membrane fluidity to explain these observations have been suggested [13, 21, 39].

In contrast to other eukaryotes, nematodes make use of structures other than plasma membranes, eggshells for eggs, and cuticles for later stages, which provide an additional external protective layer [35, 50]. Eggshells and cuticles are highly complex structures. Eggshells are thirty times thicker than cell membranes and have a different biochemical composition. They comprise three layers: an external vitelline layer, a medial chitinous layer, and a basal lipid/protein layer [35]. Membrane proteins have been identified in these barriers. They include active Pgp-like pumps, which are involved in the transport and elimination of lipophilic drugs, such as the anthelmintic ivermectin [46, 47].

In this study, we examine the relationship between Pgp number and activity, resistance to anthelmintics, and eggshell cholesterol content and fluidity in *Haemonchus contortus* nematode eggs showing different degrees of resistance to anthelmintics. The effects on fluidity of changes in the cholesterol content of eggshells were estimated by measurement of fluorescence anisotropy (FA) which is inversely proportional to membrane fluidity [39, 74, 75]. The consequences of these changes on Pgp activity were assessed by specific mAb staining, measurements of rhodamine 123 (R123) transport, and resistance to anthelmintics (thiabendazole). Four *H. contortus* (Hc) isolates were studied: two susceptible (HcS) and two resistant (HcR) isolates.

## Materials and methods

### Ethics

All experiments were conducted in accordance with EU guidelines and French regulations (Directive 2010/63/EU, 2010; Rural Code, 2018; Decree No. 2013-118, 2013). All experimental procedures were evaluated and approved by the Ministry of Higher Education and Research (APAFIS#00219.02 Notification-1). Procedures involving sheep were evaluated by the ethics committee of the Val de Loire (CEEA VdL, committee number 19) and took place at the INRAE Experimental Infection Platform PFIE (UE-1277 PFIE, INRAE Centre de Recherche Val de Loire, Nouzilly, France, https://doi.org/10.15454/1.5535888072272498e12).

### Parasites and animals

**Figure 1:**
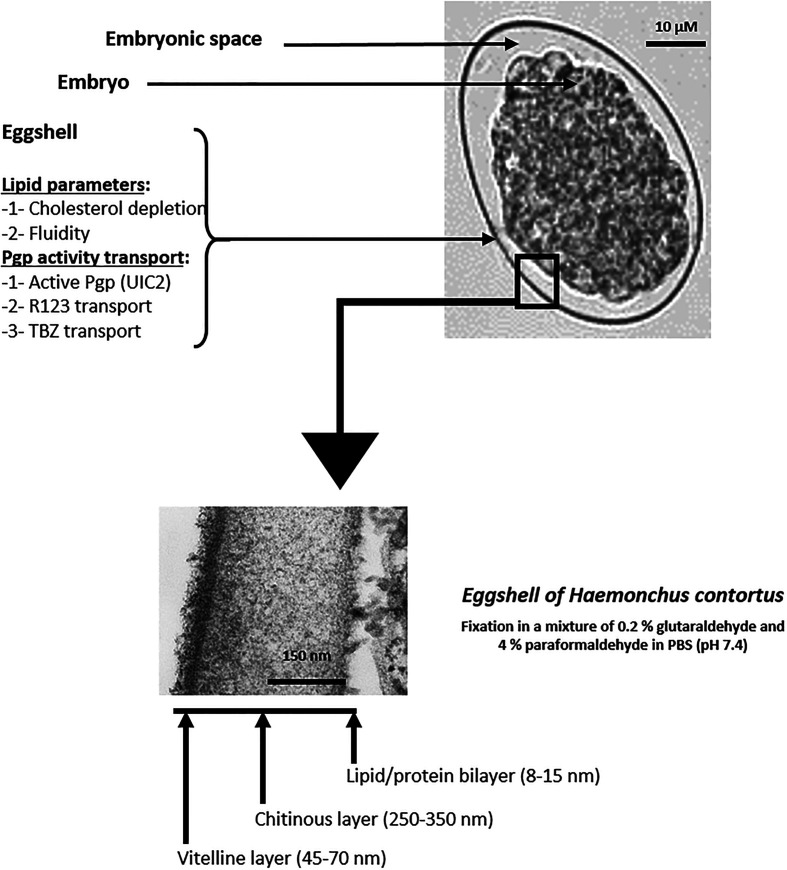
Biological model: *Haemonchus contortus* egg and eggshell.

Four *H. contortus* (Hc) isolates were studied: two susceptible (HcS) isolates (HcS-WB for “Weybridge”, UK and HcS-Ca for “Canada”) and two resistant (HcR) isolates (HcR-G for “Guadeloupe” resistant to benzimidazoles and ivermectin and tolerant to moxidectin and HcR-WR for “White River”, South Africa, (resistant to benzimidazoles and ivermectin). Eggs (Fig. 1) were isolated from faeces. Three-month-old male “Ile de France-Charolais” lambs fed with hay and cereals were infected with 6000 *H. contortus* infective larvae (L3) from each isolate. The experiments comply with the current French laws on animal experimentation.

### Cholesterol depletion

Methyl-beta-CycloDextrin (MβCD, Sigma-Aldrich, Saint-Quentin, France) was used to deplete cholesterol from eggs. In solution, the MβCD cavity is occupied by water molecules. This creates a state of unfavourable energy due to polar–apolar interactions. Water molecules are therefore easily replaced by less polar molecules, such as membrane cholesterol, toward which MβCD has strong affinity. In addition, the cholesterol dissolved in priority in the hydrophobic cavity of the MβCD [15, 51]. Eggs were incubated four times for 1 h each with shaking process, in 2.25 mM MβCD dissolved in deionised water [66]. The eggs were washed with deionised water between incubations.

Egg viability after MβCD treatment was checked using egg hatch assays. After the last washing, 2500 eggs were incubated with 150 μL of deionised water for 48 h at 22 °C.

Cholesterol and phospholipid concentrations in eggs were estimated before and after MβCD treatment [66]. Total lipids were extracted from 200,000 eggs ground in chloroform/methanol solution (v/v; VWR International, Pessac, France). Total cholesterol concentration was determined by the cholesterol oxidase method, RTU Kit, BioMérieux, Marcy-l’Étoile, France.

The total phospholipid concentration was determined by the phospholipid hydrolase method (PAP150 Kit, BioMérieux, Marcy-l’Etoile, France). The intensity of pink colouration, after enzymatic transformation of phospholipids in quinoneimine, was measured by absorbance at 505 nm. The phospholipid concentration (ng/egg) was deduced from a calibration curve using a reference phospholipid solution.

### Estimation of membrane fluidity

Membrane fluidity was estimated by fluorescence anisotropy (FA) measurements after labelling eggs with the fluorescent lipophilic probe 1,6-diphenyl-1,3,5-hexatriene (DPH, Sigma-Aldrich, Saint-Quentin, France). This probe was readily incorporated into the membrane bilayers. FA is inversely proportional to membrane fluidity. FA values close to 0.362 and more correspond to a highly organised medium and thus to very low fluidity, while FA values close to 0.100 correspond to a very fluid lipid organization, and thus to high membrane fluidity.

Optimal contact time and DPH concentration for analysing egg membrane fluidity were determined in preliminary experiments using the HcR-G isolate. DPH concentrations from 1 × 10^−7^ M to 1 × 10^−4^ M diluted in PBS were prepared from a DPH 2 × 10^−3^ M stock solution in tetrahydrofuran (THF; final, Sigma-Aldrich, Saint-Quentin, France). A bell-shared curve was obtained for anisotropy plotted against DPH concentration, with a maximum at 1 × 10^−6^ M. To measure the anisotropy into eggshell, the optimal fluorescent DPH concentration at 1 × 10^6^ M was chosen for this study and as described in other cellular models. This concentration, used in other cellular models, was chosen in subsequent experiments. Four contact times (15, 30, 45, and 60 min) were compared for two DPH concentrations (1 × 10^−6^ M and 1 × 10^−4^ M). For 15 and 30 min contact times, anisotropy was unchanged, but lower anisotropy values were obtained for 45 and 60 min contact times.

We incubated 30,000 eggs in 3 mL of a fresh dilution of DPH in PBS before and after MβCD treatment. In these conditions, the probe was found primarily in the egg membrane as it did not have enough time to diffuse more widely. A temperature of 20 °C was used as this is the optimum temperature for parasite development *in vitro.* Additionally, this was the temperature used for the various treatments previously shown to affect parasite resistance.

**Figure 2:**
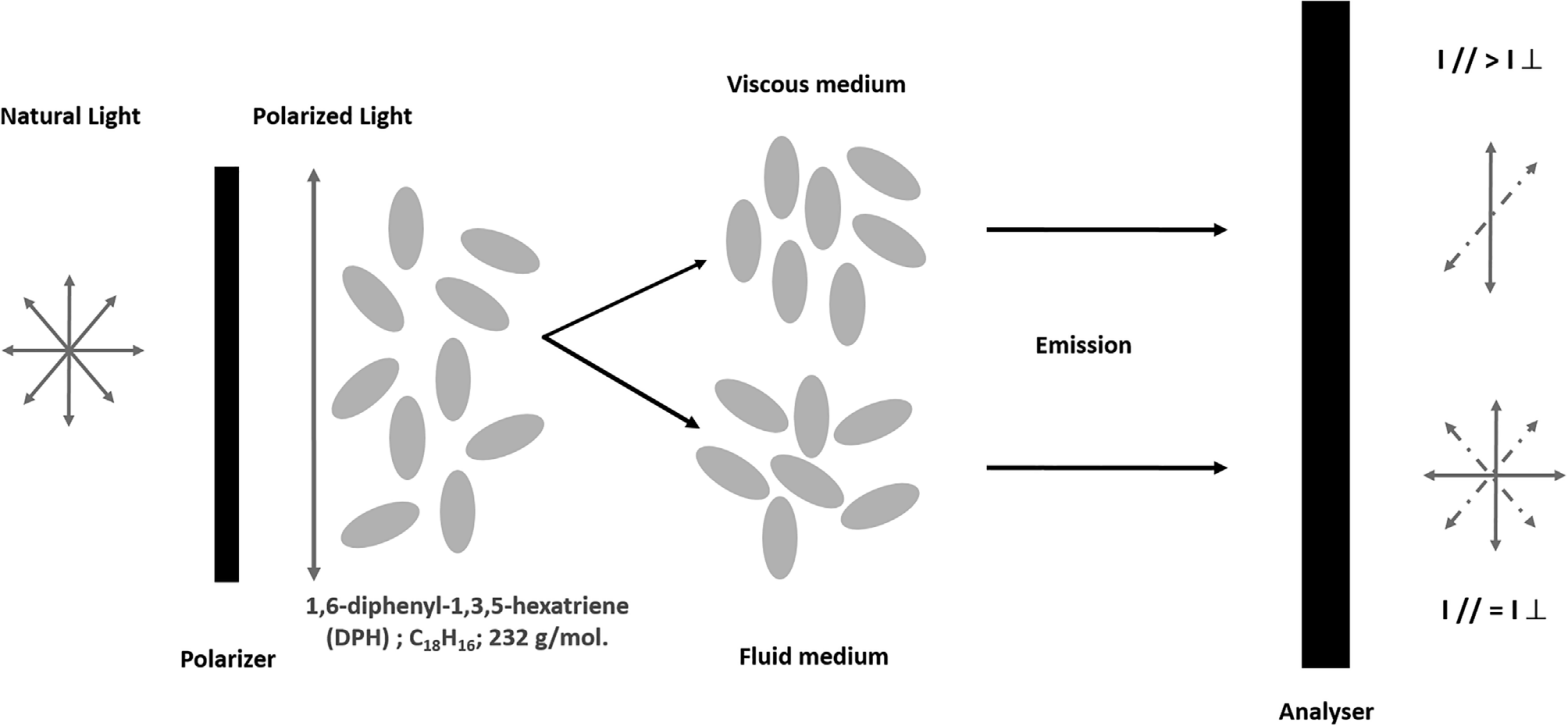
Physical principle of anisotropy measures (membrane fluidity) after incorporation of the 1,6-diphenyl-1,3,5-hexatriene (DPH) probe with a dual channel PTI Quanta Master Spectrofluorimeter (PTI, Monmouth Junction, NJ, USA). *I*//: parallel intensity; *I*⊥: perpendicular intensity.

The fluorescence anisotropy regression coefficient (*r*) was calculated from fluorescence intensity measurements with a dual channel PTI Quanta Master Spectrofluorimeter (PTI, Monmouth Junction, NJ, USA), through crossed polarizing filters. Felix software^®^ provided a macro-command for the calculation of anisotropy. The anisotropy coefficient *r* was calculated as follows:

r=(I//-gI⊥)/(I//+2gI⊥)

where parallel (*I*//) and perpendicular (*I*⊥) intensity were the respective emission fluorescence intensities through parallel and perpendicular filters to a vertical polarised excitation beam (*λ*_excitation_ = 365 nm and *λ*_emission_ = 430 nm). The g factor is a correction factor calculated before each batch of measurements (Fig. 2). With the number of eggs used in each test, no significant light scattering occurred due to autofluorescence of eggs in PBS solution [21, 39, 74, 75].

### Pgp activity assays

#### Identification of active Pgp

The presence of Pgp in active conformation was determined by UIC2 mAb staining (Immunotech, Marseille, France), estimated by flow cytometry using a MoFLo™ cell sorter (Beckman Coulter, Fort Collins, CO 80825, USA) before and after 2.25 mM MβCD treatment. The UIC2 mAbs recognise an epitope associated with a specific active Pgp conformation induced by drugs. Briefly, eggs were pre-treated with PBS plus BSA (2 mg/mL) and decanted for 10 min. They were further washed in 1 mL PBS. The eggs were stained for 90 min at room temperature by adding 35 μL of pure UIC2 mAb coupled with phycoerythrin (UIC2-PE). They were washed twice with 3 mL PBS and suspended in 1 mL PBS. The intensity of orange fluorescence was immediately measured by flow cytometry with a 580/30 nm band pass filter. Control eggs were similarly treated with isotypic IgG2a mAbs coupled with PE (IgG2a-PE, U7.27 clone, Immunotech, Marseille, France). The fluorescence means were expressed in arbitrary units (au) for the four isolates. The positive egg populations were obtained by histogram subtractions [29, 30, 32, 34].

#### Transport activity

Xenobiotic transport was determined by rhodamine 123 accumulation (R123 Sigma-Aldrich, Saint-Quentin, France), a fluorescent substrate specific for Pgp pumps, before and after MβCD treatment. R123 absorptive transport occurs primarily by the paracellular route, whereas R123 secretory transport involves influx across membrane mediated solely by a saturable process followed by apically directed efflux via Pgp (fixation on the *R* site). R123 is therefore a good model for characterising the transport of drugs such as anthelmintics (such as thiabendazole, levamisole, and ML) by Pgp.

In all, 30,000 eggs were incubated with 1 mL of R123 (0.5 μg/mL) at room temperature for 30 min and then washed with deionised water. The intensity of green fluorescence was immediately measured by flow cytometry on a MoFLo™ cell sorter (Beckman Coulter, Fort Collins, CO 80825, USA), with a 530/40 nm band pass filter. The results were expressed in arbitrary units (AU) calculated as the difference between the fluorescence of eggs without R123 and the fluorescence of eggs stained with R123, thus eliminating any native green fluorescence, which differed between isolates [12, 30, 31, 66, 69].

### Resistance to thiabendazole by egg hatch assays after MßCD treatment

A total of 2500 eggs/sample were treated, as described previously. The eggs were incubated for 48 h at 22 °C with concentrations of thiabendazole ranging from 0.02 to 0.08 μg/mL for the susceptible isolates, and from 0.24 to 1.26 μg/mL for the resistant ones [4, 7, 29]. Hatching rates were compared to those of control eggs treated with deionised water or thiabendazole only.

### Statistical analyses

Three replicates were performed for each treatment and for each factor studied. Statistical analyses were performed using GraphPad Prism software, version 5.0 (GraphPad, San Diego, CA, USA). A two-way ANOVA analysis was performed to show the effects of the treatments on the measured parameters, taking into account the parasitic isolate effect. In parallel, non-parametric statistical tests (Mann–Whitney *U* tests) were carried out, followed by Bonferroni tests. Principal component analysis (PCA) and linear regressions were performed using XL*stat* software, version 7.5.2. (Addinsoft, Paris, France).

## Results

### MβCD treatment altered cholesterol content of eggs

MβCD treatment had no toxic effects on parasite development for all isolates (Table 1).

**Table 1:** Hatching rates of eggs in water (control) or after MβCD treatment (2.25 mM). The MβCD treatment had no toxic effect. Percent hatching rates (means of three egg hatch assays) of treated eggs weighted according to the percent hatching rate in control samples.

	HcS-WB	HcS-Ca	HcR-WR	HcR-G
Untreated eggs	100	100	100	100
MβCD (2.25 mM)	106.5	98.7	105.3	110.5

HcS-WB: *Haemonchus contortus* susceptible Weybridge, HcS-Ca: *Haemonchus contortus* susceptible Canada, HcR-WR: *Haemonchus contortus* resistant White River, and HcR-G: *Haemonchus contortus* resistant Guadeloupe.

**Figure 3:**
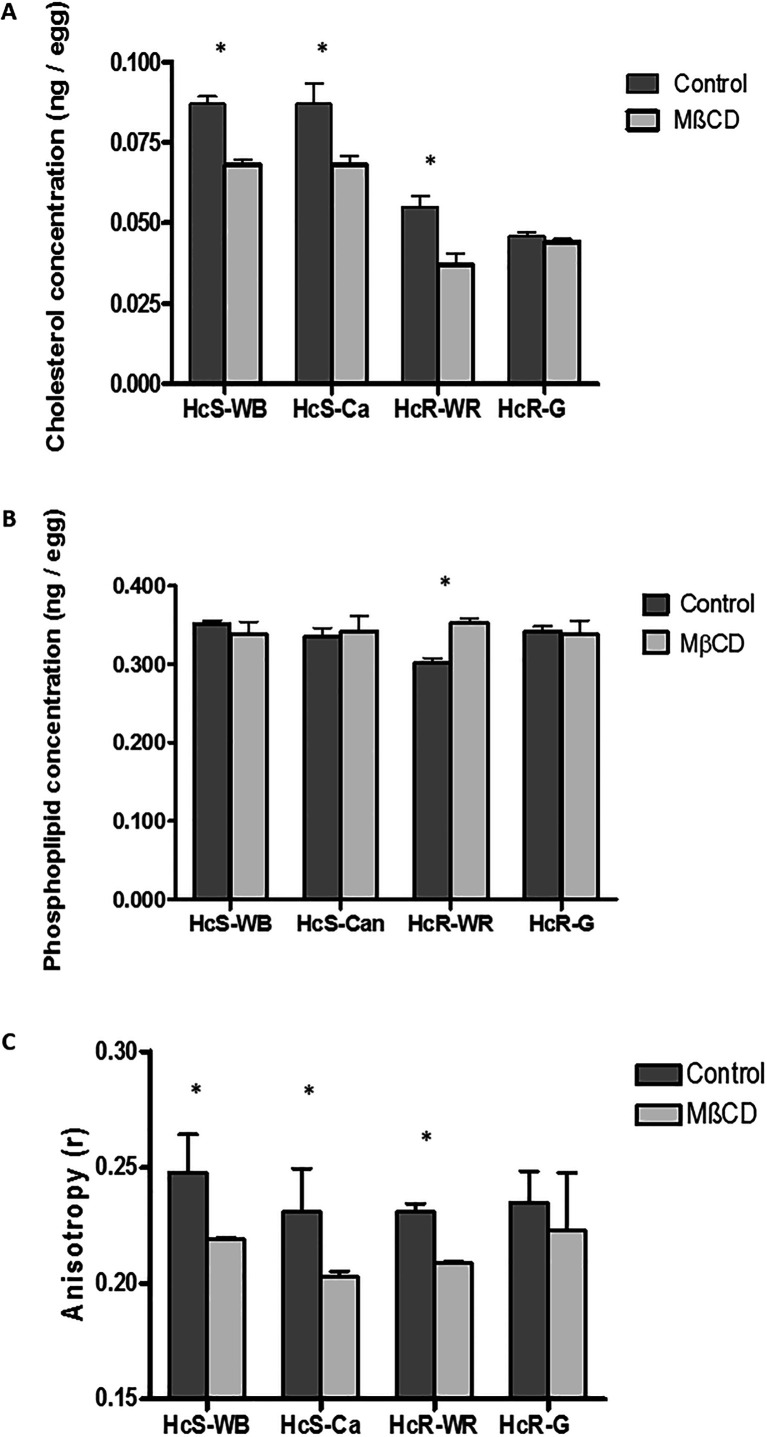
(A) Measurement of cholesterol content in nematode eggs before and after MβCD (2.25 mM) treatment. The cholesterol content of the two susceptible isolates was higher than that of the two resistant isolates (*p* < 0.05). Treatment of eggs significantly decreased the cholesterol concentration in eggs, except for the HcR-G isolate (*p* < 0.05). (B) Measurement of phospholipid content in nematode eggs before and after MβCD (2.25 mM) treatment. The phospholipid content was not modified by the MβCD treatment. (C) Measurement of anisotropy after alterations in eggshells. MβCD treatment decreased the anisotropy significantly in HcS-WB, HcS-Ca, and HcR-WR isolates (*p* < 0.05). Means of lipid concentrations (*M* ± *SD* for three measurements). *Significant effect (*p* < 0.05). Symbols: (grey rectangles) control eggs and (black rectangles) MβCD treatment. HcS-WB: *Haemonchus contortus* susceptible Weybridge, HcS-Ca: *Haemonchus contortus* susceptible Canada, HcR-WR: *Haemonchus contortus* resistant White River, and HcR-G: *Haemonchus contortus* resistant Guadeloupe.

Before treatment with MβCD, cholesterol content was significantly higher in the two susceptible isolates than in the two resistant isolates (Fig. 3A, *p* < 0.05). MβCD treatment significantly decreased the cholesterol concentration of eggs for the HcS-WB, HcS-Ca, and HcR-WR isolates (*p* < 0.05), but the effect was not significant for the HcR-G isolate (means of lipid concentration ± *SD* for three measurements). After the MβCD treatment, total phospholipid content was not modified significantly for the HcS-WB, HcS-Ca, and HcR-G isolates, except for the HcR-WR (Fig. 3B, *p* < 0.05). The phospholipid concentrations before treatment were similar between the four isolates.

### Egg anisotropy depended on changes in the lipid content

Before treatment with MβCD, egg anisotropy was significantly higher in the susceptible HcS-WB isolate than in the other three isolates (*p* < 0.05). MβCD treatment significantly decreased fluorescence anisotropy (FA) of eggs for the HcS-WB, HcS-Ca, and HcR-WR isolates (*p* < 0.05), but the effect was not significant for the HcR-G isolate (Fig. 3C).

### Pgp activity

#### The number of “active” Pgps after cholesterol depletion

**Figure 4:**
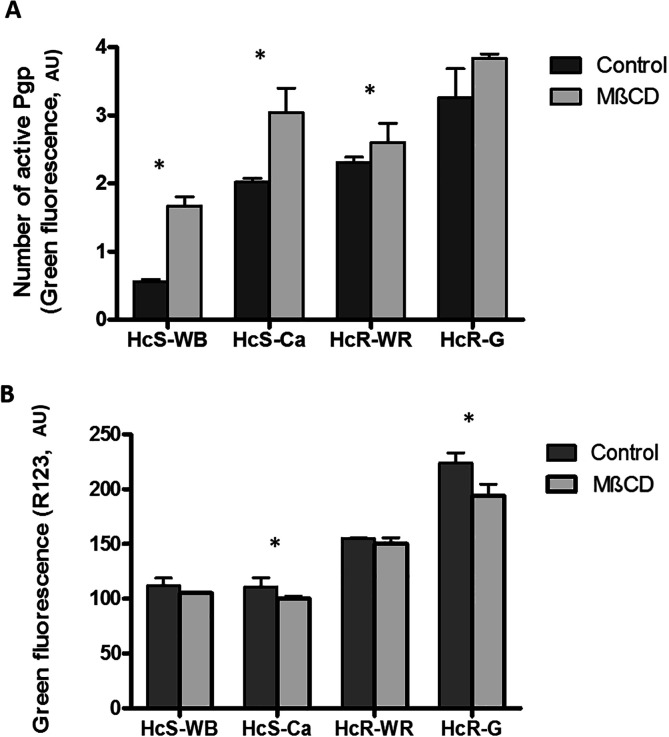
Quantification and measurement of Pgp activity in nematodes after MβCD (2.25 mM) treatment. (A) Determination of active Pgp in nematode eggs before and after MβCD (2.25 mM) treatment by UIC2 mAb staining. MβCD treatment increased the UIC2 staining significantly for the HcS-WB, HcS-Ca, and HcR-WR isolates after MβCD (2.25 mM) treatment. Mean fluorescence intensity (*M* ± *SD* for three measurements). *Significant effect (*p* < 0.05). (B) Untreated susceptible nematode isolates accumulated significantly less R123 than untreated resistant isolates (*p* < 0.05). Significant difference of R123 accumulation between susceptible and resistant isolates (*p* < 0.05). MβCD treatment decreased R123 accumulation, significantly for the HcS-Ca and HcR-G isolates (*p* < 0.05). Mean fluorescence intensity (*M* ± *SD* for three measurements) was estimated from the difference between the native green fluorescence of eggs and that of eggs stained with R123. *Significant effect (*p* < 0.05). Symbols: (grey rectangles) control eggs and (black rectangles) MβCD treatment. HcS-WB: *Haemonchus contortus* susceptible Weybridge, HcS-Ca: *Haemonchus contortus* susceptible Canada, HcR-WR: *Haemonchus contortus* resistant White River, and HcR-G: *Haemonchus contortus* resistant Guadeloupe.

Untreated susceptible nematode isolates were significantly less stained by UIC2 staining than untreated resistant isolates (Fig. 4A, *p* < 0.05). MβCD treatment increased UIC2 staining significantly for the HcS-WB, HcS-Ca, and HcR-WR isolates (Fig. 4A, *p* < 0.05).

#### Pgp activity (efflux) after cholesterol depletion

Untreated susceptible nematode isolates accumulated significantly less R123 than the untreated resistant isolates (Fig. 4B, *p* < 0.05). The MβCD treatment only significantly decreased R123 accumulation in the HsC-Can and HcR-G isolates (Fig. 4B, *p* < 0.05).

### Resistance to thiabendazole increased after cholesterol depletion

**Figure 5:**
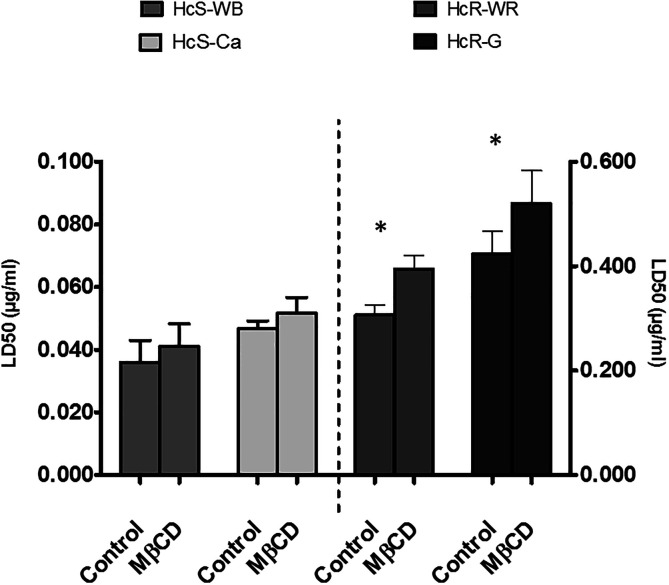
Effect of methyl-β-cyclodextrin (MβCD, 2.25 mM) on resistance to thiabendazole (lethal dose 50% or LD_50_), for each isolate. MβCD treatment increased the LD_50_ of thiabendazole (TBZ) but the effect was significant only for the two resistant isolates (*p* < 0.05). The values reported are means ± *SD* of three replicates. *Significant effect (*p* < 0.05). Symbols: (grey rectangles) control eggs and (black rectangles) MβCD treatment. HcS-WB: *Haemonchus contortus* susceptible Weybridge, HcS-Ca: *Haemonchus contortus* susceptible Canada, HcR-WR: *Haemonchus contortus* resistant White River, and HcR-G: *Haemonchus contortus* resistant Guadeloupe.

MβCD treatment increased the 50% lethal dose (LD_50_) of thiabendazole (TBZ) (Fig. 5) for the four isolates, but the effect was significant only for the two resistant isolates (HcR-WR and HcR-G, *p* < 0.05; Fig. 5).

### Multi-parametric analyses of Pgp activity

Principal component analysis (PCA) enabled us to establish a relationship between the different parameters. The Bartlett sphericity test rejects the null hypothesis of the absence of correlation between the variables (*p* < 0.0001).

Correlation analyses (Pearson test, Table 2) identified the following relationships:

- UIC2 staining, R123 accumulation and TBZ resistance are significantly correlated to cholesterol content of eggs (*p* respectively <0.04, <0.03 or <0.004);
- the number of active Pgps was significantly correlated with R123 accumulation (*p* < 0.005) and TBZ resistance (*p* < 0.008);
- R123 accumulation was significantly correlated with TBZ resistance (*p* < 0.007);
- no correlation was found between anisotropy and the four other parameters.

**Table 2:** Matrix of correlation of five parameters (cholesterol content, anisotropy, R123 accumulation, UIC2 staining, and resistance to thiabendazole [TBZ]) obtained by principal component analysis for each isolate.

Parameters	Cholesterol content	Anisotropy	UIC2 staining	R123 accumulation	Resistance to TBZ
Cholesterol content	1				
Anisotropy	0.406	1			
UIC2 staining	**−0.761 (a)**	−0.207	1		
R123 accumulation	**−0.738 (a)**	0.202	**0.876 (a)**	1	
Resistance to TBZ	**−0.895 (a)**	−0.063	**0.844 (a)**	**0.906 (a)**	1

a: Significant effect (*p* < 0.05).

**Figure 6:**
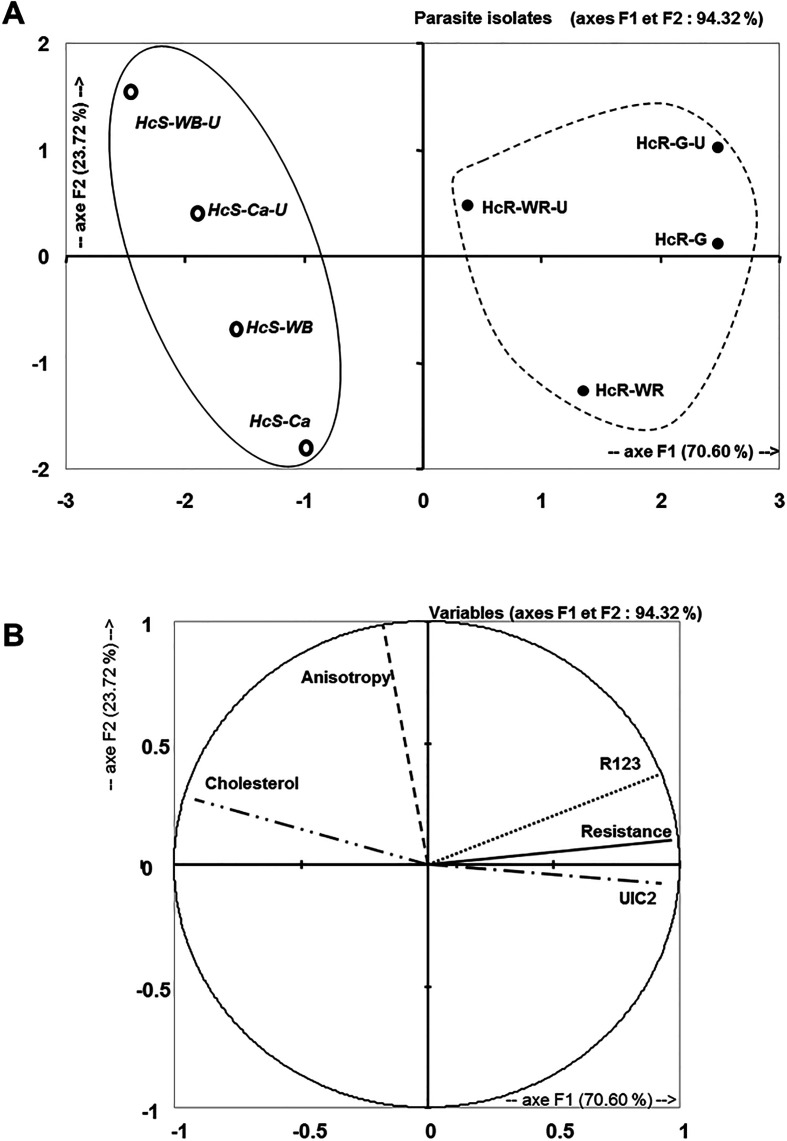
Multiparametric analyses of Pgp activity and the lipid environment in *Haemonchus contortus* nematode eggs before and after MβCD (2.25 mM) treatment. HcS-WB: *Haemonchus contortus* susceptible Weybridge, HcS-WB-U: *Haemonchus contortus* susceptible Weybridge Untreated, HcS-Ca: *Haemonchus contortus* susceptible Canada, HcS-Ca-U: *Haemonchus contortus* susceptible Canada Untreated, HcR-WR: *Haemonchus contortus* resistant White River, HcR-WR-U: *Haemonchus contortus* resistant White River Untreated, HcR-G: *Haemonchus contortus* resistant Guadeloupe, and HcR-G-U: *Haemonchus contortus* resistant Guadeloupe Untreated.

Figure 6A shows the distribution of isolates and the relationships between parameters that were explained at 94% by two axes (F1 and F2). The F1 axis is mainly linked to the cholesterol content, the number of active Pgps, R123 transport, and TBZ resistance. Cholesterol content varied in a way opposite to the other three parameters. The anisotropy was linked to the F2 axis. The F1 axis thus allowed us to distinguish two groups, resistant isolates and susceptible isolates, while the F2 axis separated the control group from the group treated with MβCD. The cholesterol content, the number of active Pgps, and R123 accumulation were highly discriminant variables for each isolate (Fig. 6B).

Several significant linear regressions were established (Table 3) between cholesterol and either Pgp activity (UIC2 or R123 accumulation) or TBZ resistance, and between Pgp activity (UIC2 or R123 accumulation) and TBZ resistance.

**Table 3:** Relationships between cholesterol content and number of Pgps in the active conformation (UIC2 antibodies), Rhodamine 123 (R123) transport, and resistance to thiabendazole (TBZ) in *Haemonchus contortus* eggs independently of MβCD treatment.

*X*	*Y*	Regression	*df*	*r*	*p*<
Cholesterol content	Anisotropy	*Y* = 0.5354*x* − 0.0589	7	1	NS
Cholesterol content	R123 accumulation	*Y* = −0.0003*x* + 0.1061	7	1	0.05
Cholesterol content	UIC2 staining	*Y* = −0.0211*x* + 0.2083	7	1	0.05
Cholesterol content	Resistance to TBZ	*Y* = −0.084*x* + 0.0806	7	1	0.01
R123 accumulation	Resistance to TBZ	*Y* = 0.0041*x* − 0.3557	7	1	0.01
R123 accumulation	UIC2 staining	*Y* = 0.0131*x* + 5.0653	7	1	0.01
Resistance to TBZ	UIC2 staining	*Y* = 2.8585*x* + 6.3059	7	1	0.01

NS: non-significant; *r*: regression coefficient; *df*: statistic data corresponding to the degree of freedom.

## Discussion

We previously suggested that a reduction in cholesterol concentrations may lead to changes in the organisation of membrane lipids and possibly affect the diffusion of lipophilic molecules such as R123 or anthelmintics in eggshells. Consistent with this hypothesis and with the usual observations made on plasma membranes, we observed in the present study an increase in eggshell fluidity after cholesterol depletion by MβCD in both susceptible and resistant nematodes. Therefore, cholesterol depletion seems to modify the organisation of lipid eggshells. Cholesterol depletion induces an increase in the fluidity of the eggshell of nematode, like in other conventional membrane systems [15, 26, 82].

In the present work, and for the first time, we showed that resistance of nematodes to anthelmintics increased following cholesterol depletion, which could be attributed to fluidification of the eggshell and an increase in Pgp activity. We investigated here cellular and molecular interactions between (1) cholesterol concentrations in eggshells, (2) membrane fluidity, (3) active Pgp estimated by staining with UIC2 mAbs, (4) efflux transport by measuring the accumulation of a specific Pgp fluorescent substrate (R123), and (5) resistance to anthelmintics with thiabendazole. A very strong relationship between the five parameters studied shows a very clear differentiation between susceptible isolates and resistant isolates. Therefore, the resistance state can be defined by the following parameters: cholesterol (biological membranes)/UIC2 (active Pgp)/R123 (Pgp activity). This relationship between these parameters has been observed in other nematode species (*Caenorhabditis elegans* and *Cylicocyclus elongatus*) and other pathogens such as bacteria and fungi [6, 28, 45, 48, 49, 66]. This study was the first to measure membrane fluidity in nematodes and to establish relationships with cholesterol content, and confirmed the results obtained in other eukaryote models [20, 39, 54, 81].

We recently found that modulation of Pgp activity in nematodes can be obtained by approaches very similar to those used for other eukaryote models [32, 33, 66]. Studies on vertebrate cells showed new means for the modulation of Pgp activity after modifications of cholesterol concentrations that alter the membrane environment. The experimental change in cholesterol content was obtained using a cholesterol acceptor, methyl-β-cyclodextrin. β-cyclodextrins have high affinity for lipids [15]. Moreover, the methyl form (MβCD) preferentially extracts cholesterol from membrane cells [15, 80, 81]. We confirmed that cholesterol depletion by MβCD treatment (2.25 mM MβCD concentration four times over 60 min) did not alter the viability of *H. contortus* eggs. However, it altered their cholesterol content, the first parameter, as previously described [66]. The depletion was enough to change the total cholesterol content of eggs without any toxic effect on egg embryonation. This effect was similar to that obtained with a higher concentration, i.e. 75 mM for a shorter contact time, i.e. 10 min [66].

The second parameter modified after MβCD treatment is membrane fluidity, estimated by anisotropy. Changes in the biophysical properties of eggshells were evaluated as described for other models, by measuring steady-state anisotropy with a fluorescent probe, 1,6-diphenyl-1,3,5-hexatriene (DPH) incorporated into the eggshells. In vertebrate cells, DPH incorporates into the hydrocarbon core of membrane bilayers [74, 75]. Despite the complexity of the *H. contortus* model, the values obtained for eggshell anisotropy and their variations with cholesterol concentrations were similar to those observed in vertebrate cells. In *H. contortus*, we showed that the embryonation of eggs increases membrane fluidity [67]. The increase in eggshell fluidity observed during parasite development reflects changes in the organisation of lipids in the membranes, and affects the subcellular distribution of anthelmintics and their access to Pgp, thereby increasing resistance. In untreated eggs and in the total absence of embryonation, fluidity is significantly lower in the eggshells of susceptible isolates than in those of resistant isolates. In untreated and embryonated eggs, isolates did not differ significantly in eggshell fluidity or cholesterol content, as previously shown. The effect of depletion was thus less marked than that of embryonation [67]. The advantage was better controlled testing conditions. The lipid content of eggs during embryonation varied and depended on the isolate. Variations in membrane fluidity thus depend on a native difference in the eggshells (lipid composition), on the efficacy of MβCD treatment, and on egg embryonation. In this work, our four parasite isolates responded significantly to MβCD treatment on lipid measured parameters, except for the HcR-G isolate. Our hypothesis is that the sterol lipid composition of the HcR-G eggshell is different from the other three isolates and MβCD did not have the same affinity for the sterols present in the HcR-G eggshell.

Alongside changes in the eggshell after MβCD treatment, it is important to analyse the impact of treatment on the last three parameters: (i) active Pgp, (ii) the activity of transport by Pgp, and (iii) the relationship between the efflux pump and TBZ resistance [73, 74]. An increase in membrane fluidity induced by MβCD changed structural conformation of Pgps. Configuration of the membrane Pgp changes from active to very active conformation according to ATP level in the cell and alteration of lipid membranes [1, 16, 61–63]. For this last point, we showed that cholesterol depletion activates efflux pumps (Pgps).Moreover, the concentrations of membrane cholesterol goes through an optimal for the active form of Pgps [55, 68]. When Pgps are most active (optimal efflux), this activation is directly related to an increase in the transport activity of the antiparasitic, but also to an increased affinity for specific substrates such as R123 or thiabendazole. Changes in the cholesterol content of other cellular systems have been shown to affect: (a) their affinity for the substrate of transmembrane proteins such as hormonal receptors [38] or (b) the transduction of the intracellular signals [19, 43]. In our experimental conditions, it seems that the mechanism is more likely due to a modulation of transport. We hypothesize that TBZ, a hydrophobic compound, diffused passively through lipid-rich membranes. To mimic the passive diffusion and efflux exchange of TBZ, Rhodamine R123 seems to be the right candidate. It possesses similar physicochemical properties (lipophilic molecule) compared to anthelmintics and has a Pgp binding site on the *R* site [12, 18]. The flow cytometric assays on the fluorescence of nematode eggs resulting from the contact with R123 allowed us to observe this mechanism more directly. Nevertheless, only a small amount of R123 is taken up passively and this process is very slow. Therefore, the fluorescence of eggs after contact with R123 was mainly representative of the activity of Pgp [31, 66]. The intensity of green fluorescence decreased significantly after MβCD for the four isolates. As a result, a decrease in fluorescence after MβCD treatment might be attributed to stimulated Pgp activity resulting from a decrease in cholesterol content. R123 native transport increased with resistance in *H. contortus* isolates. Differences between susceptible and resistant isolates have mostly been attributed to the presence of higher amounts of Pgp in the resistant isolates, leading to the binding of larger numbers of R123 molecules than in susceptible isolates, such described in Kerboeuf et al. [34]. A final point that could impact the function and the regulation of Pgp in nematodes is the presence of different Pgp isoforms. In *H. contortus*, several Pgp isoform genes were identified such as Hco-pgp-3, Hco-pgp-9.2, Hco-pgp-11, and Hco-pgp-16, specifically up-regulated in parasitic life stages, suggesting potential involvement of these Pgps in the efflux of eosinophil granule products [27]. Some Pgp isoforms were involved in anthelmintic resistance mechanisms such as MDR1 or Pgp-1 [22–24], like in other pathogens or cellular lines [28, 52], and other Pgps such as Pgp-3 (MDR3) implicated in lipid transport [10, 11, 77, 78]. In our study, the different isolates may possess different pump isoforms (amount of protein and gene expression) with different susceptibilities towards depletion. The relationship between Pgp isoforms and membrane lipids could thus modulate Pgp activity, particularly those associated with resistance to anthelmintics, as demonstrated by Riou et al. to resistance of thiabendazole [68].

It can therefore be suggested that the solubilisation of lipophilic molecules is, as a consequence, altered and that cholesterol depletion may favour an increase in Pgp activity, accompanied by a decreased in R123 accumulation in eggs. It is difficult to determine the relative contributions of changes in the solubilisation of lipophilic molecules (R123 or anthelmintics) and transport by cellular pumps (Pgp). The mechanisms described here for the modulation of R123 transport by cholesterol, if applied to the transport of anthelmintics in nematodes may account, at least in part, for the observed changes in resistance to anthelmintics. Anthelmintics must be solubilised in membrane lipids, in which they accumulate, before they can penetrate eggs. Anthelmintics are also Pgp substrates and are eliminated by these pumps. The mechanisms of xenobiotic transport by Pgp are not fully understood, but changes in the membrane environment may be involved in regulating anthelmintic transport. The roles of the various components of lipophilic molecule transport systems (passive diffusion, active influx, and active efflux) need to be investigated further, as well as the role and production of lipids in nematodes. This knowledge may therefore make it possible to identify new targets for anthelmintics, like other targets described in recent research in order to counter multiple resistance [8, 14, 46, 53, 57, 79].

## Conclusion

Surprisingly, eggshells have certain biophysical properties common with the plasma membrane of vertebrate cells, but a more complex structure and biochemical composition. Eggshells appear to be more than a simple physical barrier and resemble membranes in having active biological properties. The membrane lipid composition of eggshells seems to have a significant effect on the regulation of anthelmintic transport in nematodes.

Fluidity is a complex parameter depending on many factors, including lipid composition (sterols, phospholipids, unsaturated fatty acids, etc.), and the presence of membrane proteins such as Pgp. A reduction in cholesterol content in the eggshell increased the number of active Pgps and altered TBZ solubilisation into the eggshell, and thus changed resistance to anthelmintics. The nematode egg was therefore considered a very good model for studying resistance to anthelmintics.
